# Anthropogenic ecosystem fragmentation drives shared and unique patterns of sexual signal divergence among three species of Bahamian mosquitofish

**DOI:** 10.1111/eva.12275

**Published:** 2015-07-16

**Authors:** Sean T Giery, Craig A Layman, R Brian Langerhans

**Affiliations:** 1 Marine Sciences Program, Department of Biological Sciences, Florida International UniversityNorth Miami, FL, USA; 2 Department of Biological Sciences and W.M. Keck Center for Behavioral Biology, North Carolina State UniversityRaleigh, NC, USA; 3 Department of Applied Ecology, David Clark Labs, North Carolina State UniversityRaleigh, NC, 27695, USA

**Keywords:** coloration, divergent evolution, *Gambusia*, historical contingency, parallel evolution, rapid evolution, sexual selection

## Abstract

When confronted with similar environmental challenges, different organisms can exhibit dissimilar phenotypic responses. Therefore, understanding patterns of phenotypic divergence for closely related species requires considering distinct evolutionary histories. Here, we investigated how a common form of human-induced environmental alteration, habitat fragmentation, may drive phenotypic divergence among three closely related species of Bahamian mosquitofish (*Gambusia* spp.). Focusing on one phenotypic trait (male coloration), having *a priori* predictions of divergence, we tested whether populations persisting in fragmented habitats differed from those inhabiting unfragmented habitats and examined the consistency of the pattern across species. Species exhibited both shared and unique patterns of phenotypic divergence between the two types of habitats, with shared patterns representing the stronger effect. For all species, populations in fragmented habitats had fewer dorsal-fin spots. In contrast, the magnitude and trajectory of divergence in dorsal-fin color, a sexually selected trait, differed among species. We identified fragmentation-mediated increased turbidity as a possible driver of these trait shifts. These results suggest that even closely related species can exhibit diverse phenotypic responses when encountering similar human-mediated selection regimes. This element of unpredictability complicates forecasting the phenotypic responses of wild organisms faced with anthropogenic change – an important component of biological conservation and ecosystem management.

## Introduction

Human-mediated ecological change represents the primary driver of contemporary evolutionary change (Palumbi 2001; Hendry et al. 2008). While we know that ecological alteration affects evolutionary futures of species and populations, we know less about the predictability or the consequences of these changes (Hendry et al. 2011; Sih et al. 2011; Barrett and Hendry 2012; Palkovacs et al. 2012). Given the striking and ongoing reduction of biodiversity caused by human activities (Dirzo and Raven 2003; Barnosky et al. 2011), these gaps in our knowledge require rapid attention if we are to devise new conservation approaches that employ evolutionary principles (Dawson et al. 2011; Carroll et al. 2014; Smith et al. 2014).

The predictability of evolutionary change has been repeatedly illustrated by parallel and convergent evolutionary patterns in a diversity of taxa (e.g., sticklebacks: Schluter and McPhail 1992; cichlids: Kocher et al. 1993; *Anolis* lizards: Losos et al. 1998; Mahler et al. 2013; guppies: Endler 1980; Reznick and Endler 1982; Iguanid lizards: Rosenblum 2006). Discovery of these shared adaptive responses to similar selection regimes has served to demonstrate natural selection as a major and predictable driver of evolutionary change (Schluter and McPhail 1993; Losos 2010). Yet, when distinct evolutionary histories or chance events interact with selection, evolutionary divergence may follow nonparallel (unique) trajectories (Gould and Lewontin 1979; Gould 1989; Taylor and McPhail 2000). Evolutionary histories can vary among lineages for reasons other than divergent selection regimes, including genetic drift (Fisher 1930; Lande 1981), founder effects (Mayr 1942; Nei et al. 1975), and mutation-order selection (Mani and Clarke 1990; Rundell and Price 2009; Schluter 2009). These different agents of evolutionary change can generate interpopulation differences in genetic architecture that in turn affect the magnitude and trajectory of evolution, even when selection pressures are similar (Travisano et al. 1995; Schluter 1996; Taylor and McPhail 2000; Langerhans and DeWitt 2004; Blount et al. 2008; Langerhans 2010; Rosenblum and Harmon 2011; Kaeuffer et al. 2012).

How predictable and repeatable are human-induced phenotypic shifts in the wild? Anthropogenic environmental alteration is pervasive and global in extent (Vitousek et al. 1997; Palumbi 2001), and as a result, humans are generating similar selection pressures in human-modified environments for multiple populations within species, and for multiple species across geographic regions. We currently know little about the predictability of phenotypic divergence in response to anthropogenic change, or the extent to which different groups of organisms (i.e., different populations or species) respond to the same human-induced stressor(s) in shared or unique ways. When faced with similar and severe human-induced impacts, do closely related species generally exhibit similar phenotypic responses, or do species-specific responses predominate? Answering this question is critical for understanding human-mediated evolutionary change in the wild and how to manage it (Hendry et al. 2011; Carroll et al. 2014; Smith et al. 2014).

In this study, we investigate patterns of phenotypic divergence within three species of Bahamian mosquitofish (*Gambusia* spp.) subjected to hydrologic fragmentation, a widespread form of anthropogenic change (Montague et al. 1987; Pringle 2001; Nilsson et al. 2005). Fragmentation of Bahamian tidal creeks, a primary habitat of Bahamian mosquitofish, results in consistent ecological change, especially the local extirpation of piscivorous fishes (Layman et al. 2004, 2007; Heinen-Kay et al. 2014; Chacin et al. 2015). Because piscivorous fishes often exert predictable negative selection pressure on conspicuous coloration in their prey (Endler 1980, 1983; Houde 1997; Martin et al. 2014; Giery and Layman 2015), we focused our investigation on divergence in mosquitofish coloration. Given reduced predation pressure in fragmented ecosystems, we predicted that populations inhabiting these tidal creek habitats would exhibit more conspicuous coloration. Because fragmentation also appeared to cause shifts in the spectral environment, a recognized agent of color divergence, we additionally examined the effects of turbidity (Seehausen et al. 1997; Dugas and Franssen 2011) and spectral composition (Endler 1991; Fuller 2002; Morrongiello et al. 2010) in driving phenotypic variation. To test our prediction, we applied a ‘shared and unique’ statistical approach (Langerhans and DeWitt 2004) that addresses two main questions: (i) Does fragmentation, a shared contemporary ecological disturbance, affect coloration in Bahamian *Gambusia*, and (ii) Are these effects consistent among three distinct *Gambusia* species?

## Methods

We conducted our study in tidal, mangrove-dominated wetlands in the Bahamas, locally known as ‘tidal creeks’. Tidal creeks comprise shallow, tidally influenced wetlands, typically fringed by red mangrove (*Rhizophora mangle*). Hydrologic flux in tidal creeks derives from tidal exchange with the ocean, as the creeks have small watersheds with karstic bedrock. Consequently, intact tidal creeks have salinities around 35 ppt, and biotic communities similar to other mangrove-dominated systems (Layman et al. 2004; Valentine-Rose et al. 2007). Major predators of Bahamian mosquitofish in these systems include redfin needlefish (*Strongylura notata*), snapper (*Lutjanus* spp.), and great barracuda (*Sphyraena barracuda*) (Layman et al. 2004; Araújo et al. 2014; Giery and Layman 2015).

**Figure 1:**
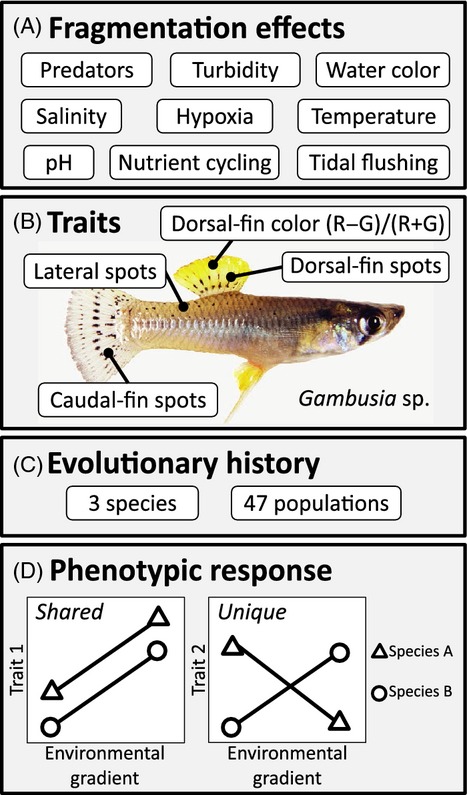
Anthropogenic hydrologic fragmentation causes myriad ecological changes to Bahamian tidal creeks (A). This study focuses on the effects of fragmentation on the coloration of an organism persisting in these altered ecosystems, the Bahamian mosquitofish (*Gambusia* spp.; B). Our study system includes replicate populations (47) representing three allopatric, sister species of Bahamian mosquitofish from the Bahama Archipelago (C). Our analysis employs a ‘shared and unique’ analytical framework to examine the effect of independent evolutionary histories (i.e., species identity) on the magnitude and trajectories of phenotypic responses to an anthropogenic environmental disturbance, tidal creek fragmentation. A shared response (bottom left inset panel) implies parallel divergence between disturbed and undisturbed environments and a unique response evidences nonparallel divergence among species (bottom right inset panel; D).

Fragmentation of Bahamian tidal creeks results from road construction, usually near creek mouths. These road blockages often lack flow-conveyance structures, such as bridges or culverts, resulting in disruption of hydrologic connectivity. By blocking tidal exchange between landward sections of tidal creeks and the ocean, fragmentation precipitates a suite of environmental changes in blocked portions of tidal creeks including the extirpation of piscivorous fishes, lower dissolved oxygen daily minima, increased daily maximum temperature, altered salinity, higher turbidity, higher pH, and increased nutrient availability (Valentine-Rose et al. 2007; Allgeier et al. 2010; Heinen-Kay et al. 2014; Chacin et al. 2015; Fig.1A). Therefore, fragmentation *per se* does not directly result in environmental change in blocked tidal creeks; rather, disruption of hydrologic connectivity induces an ecological cascade culminating in novel environmental conditions to which fragmentation-tolerant organisms, such as mosquitofish, may adapt. While the exact dates of each fragmentation event in this study are not known, most roads that bisect tidal creeks in this study were built between the 1950s and 1970s during an expansion of infrastructure associated with intensive logging and coastal development in The Bahamas.

Bahamian mosquitofish (*Gambusia* spp.) are small, live-bearing fish with short generation times (1–2 generations per year) found in a wide range of aquatic habitats in The Bahamas including tidal creeks, blue holes, and inland freshwater marshes (Downhower et al. 2000; Langerhans et al. 2005, 2007; Araújo et al. 2014; Giery and Layman 2015). Three species of Bahamian mosquitofish reside in our study area, the northern Bahama Archipelago (Heinen-Kay et al. 2014). *Gambusia hubbsi* inhabits the Great Bahama Bank, including the islands of Andros and New Providence. *Gambusia manni* also inhabits several islands of the Great Bahama Bank (not known to occur sympatrically with *G. hubbsi* in any tidal creek), including the islands of Eleuthera and Long Island. A third, unnamed species, *Gambusia* sp., appears limited to the Little Bahama Bank, including the islands of Grand Bahama and Abaco. These three species, endemic to The Bahamas, form a monophyletic group of closely related species with similar natural histories and morphologies, and are collectively referred to as Bahamian mosquitofish. Current data suggest *G. hubbsi* and *G. manni* are sister taxa, with *Gambusia* sp. diverging from the clade of *G. hubbsi* and *G. manni* approximately 1.7–4.8 million years ago, and *G. hubbsi* and *G. manni* diverging from one another approximately 1.3–2.4 million years ago (Heinen-Kay et al. 2014).

Adult male Bahamian mosquitofish express heritable variation in dorsal-fin coloration that ranges from intensely reddish orange to pale yellow (Fig.1B; Figure S1; Langerhans 2006; Martin et al. 2014; Giery and Layman 2015). Coupled with findings that show heritable female preference for brightly colored dorsal fins (Heinen-Kay et al. 2015), the conspicuous display of dorsal fins during courtship, exclusive presence in males, ontogenic elaboration, and condition dependence suggest that they are secondary sexual characters subject to sexual selection via female preference (Andersson 1994). Bahamian mosquitofish also exhibit numerous, small, black spots on their body, caudal and dorsal fins, and there is some evidence for a role of black spots in crypsis (Endler 1983) and signaling (Brooks and Caithness 1995; Horth 2003; Martin et al. 2014) in Poeciliid fishes. Previous work with *G. hubbsi* found reduced dorsal-fin coloration and body spotting in populations experiencing higher predation risk, suggesting that predation pressure can influence elaboration of these traits in Bahamian mosquitofish (Martin et al. 2014).

**Figure 2:**
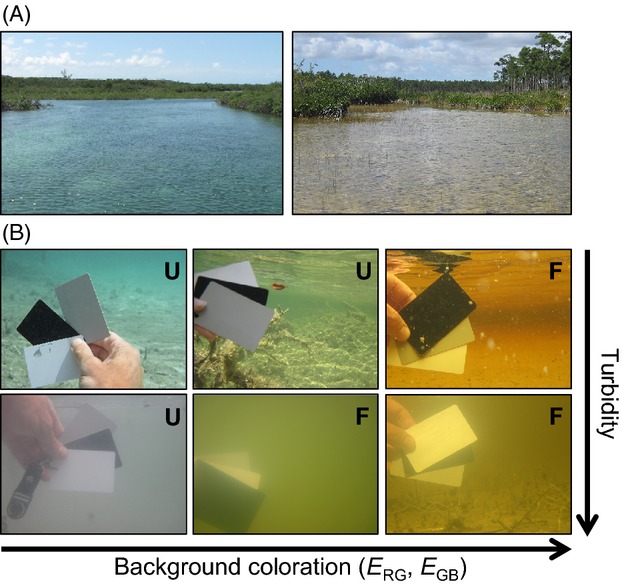
(A) Representative photographs of unfragmented (left) and fragmented (right) tidal creeks in The Bahamas (Abaco Island depicted). (B) Illustration of two major components of variation in spectral environment across unfragmented (U) and fragmented (F) tidal creeks: background color and water turbidity.

We sampled 47 *Gambusia* populations comprising all three species across the Bahama Archipelago during March and April 2010 (Fig.1C; Figure S2). Twenty-three of the collection sites were hydrologically fragmented by a road (Fig.2A). At each site, we examined 3–8 adult males for color analysis. Fish were lightly anesthetized with eugenol (clove oil), laid on their left flank, and photographed on a white background in uniform, indirect, natural light with a Canon D10 digital camera. A ruler was included in each photo for measurement of body length (BL). Exposure and white balance were manually adjusted in Adobe Photoshop CS5 using a gray card exposure standard included in each photo. We sampled color using Adobe Photoshop CS5 in the RGB color space. RGB is a commonly employed color space for quantifying coloration in vertebrates (Stevens et al. 2007). The RGB color space defines color in a three-dimensional coordinate system where high values of R indicate red, G indicate green, and B indicate blue. We then generated an index measure of signal coloration (R−G)/(R+G) following previous work by Endler (1990, 2012) and McKay (2013). This metric estimates color (hue) along an axis from red (1) to yellow (0) and green (−1), and will be referred to as RG for the remainder of the paper. RGB measures were taken from 3 × 3 pixel samples from eight points on the dorsal fin and averaged for analysis. Four readings were located on the distal portion of the dorsal fin and four from the region near the fin insertion. The location of each measure was in areas of membrane between fin rays 2,3; 3,4; 4,5; 5,6. In addition to color, the number of black spots on the caudal fin, dorsal fin, and right flank was recorded (Fig.1).

Color vision in fishes is taxonomically widespread and well developed (Loew and Lythgoe 1978; Lythgoe et al. 1994). Color vision in ecologically similar and closely related fishes suggest that Bahamian mosquitofish also possess well-developed color vision (e.g., Levine and MacNichol 1979; Archer et al. 1987; Körner et al. 2006; Watson et al. 2010). However, because RGB is not based on *Gambusia* visual systems it does not represent color as perceived by them or their predators. Determining whether the magnitude of variation detectible using our methodology is biologically relevant for our focal species would require additional information about their visual systems. Additionally, our methodology was not sensitive to UV light. However, UV coloration in Bahamian mosquitofish fins has been examined previously with spectrometry and has failed to reveal UV reflectance (Martin et al. 2014; Heinen-Kay et al. 2015).

We selected a set of environmental variables for measurement at each site based on their hypothesized roles as environmental agents likely to affect selection on coloration: predation pressure (Endler 1980, 1983; Horth 2004; Martin et al. 2014), turbidity (Seehausen et al. 1997; Dugas and Franssen 2011), and spectral environment (Endler 1991; Morrongiello et al. 2010; but see Schwartz and Hendry 2010). Fish communities were surveyed using roving diver surveys (Layman et al. 2004) and belt transects in areas where fish congregate such as mangrove fringes and pools (Faunce and Serafy 2007). Piscivore densities were calculated using the area sampled at each tidal creek, up to 1000 m^2^ (Heinen-Kay et al. 2014). Fishes classified as piscivores included snappers (*Lutjanus* spp.), needlefishes (*Strongylura* spp.), groupers (*Epinephelus* spp.), great barracuda (*S. barracuda)*, Atlantic tarpon (*Megalops atlanticus*), and jacks (*Caranx* spp.). Invertivores such as grunts (*Haemulon* spp.) and mojarra (*Gerreidae* spp.) were not classified as piscivores (Layman and Silliman 2002). Turbidity was measured in the field with an Oakton T100 turbimeter to the nearest nephelometric turbidity unit (NTU). To estimate the color of the aquatic habitat, we took underwater photos approximately 15 cm below the surface from a perspective perpendicular, and away from the shoreline at 1–4 locations within the study site and averaged for analysis (Fig.2B). In each photo, the camera settings were maintained at 1/60th sec and F 5.6 exposure. The background coloration of the environment was estimated in Adobe Photoshop CS5 with a 3 × 3 pixel sample from the background of each photo. The dominant wavelength in each site was then measured in RGB color space. Color was estimated using two RGB color metrics: RG = (R−G)/(R+G) and GB = (G−B)/(G+B). As discussed above, RG measures the variation from red (1) to yellow (0) to green (−1), while GB measures variation from green (1) to cyan (0) and blue (−1). These two metrics allow us to capture major axes of environmental color variation observed in our study sites (Fig.2).

The ‘snapshot’ nature of our sampling subjected our estimates to various extrinsic sources of environmental variation. We dealt with these sources of error in several ways. First, because radiance would be disproportionately affected by time of day or cloud cover, we restricted our analysis to parameters independent of total incident radiant flux (RGB ratios: Endler 2012). Second, we validated the temporal repeatability of our snapshot measures of water color using data available for a subset of sites (*n* = 13) following Lessells and Boag (1987). Underwater photographs from 2009, 2011, and 2012 show that our of environmental color were temporally consistent (RG *r *=* *0.45; GB *r *=* *0.58; Table S1). Previous work has demonstrated temporal repeatability of piscivore density and turbidity in Bahamian tidal creeks (Heinen-Kay et al. 2014). Thus, overall our snapshot estimates of environmental parameters should provide meaningful estimates for comparison among sites.

### Data analysis

Prior research suggests that fragmentation of tidal creeks largely results in consistent ecological changes (Layman et al. 2004; Araújo et al. 2014; Heinen-Kay et al. 2014). However, to be certain that fragmentation regime represented a consistent disturbance across the large spatial scale of this study, we evaluated the effect of fragmentation on the four putatively important drivers of variation in male coloration: water redness (hereafter *E*_RG_), water blueness (hereafter *E*_GB_), turbidity, and piscivore density across the study area. Turbidity and piscivore density were log-transformed for analysis (untransformed values in Table S2). For this analysis, we were primarily concerned with testing whether fragmentation resulted in consistent ecological change within the geographic range of each species (Figure S2). We used manova in which environmental variables served as dependent variables, and fragmentation regime, species range, and the interaction between fragmentation and species range served as independent variables. If the effect of fragmentation (shared effects of fragmentation across the species ranges) is much greater than the effect of the interaction term (geography-dependent effects of fragmentation on environmental variables), then this suggests that each species has experienced a largely similar environmental shift subsequent to fragmentation, consistent with our assumption that fragmentation regime has a shared environmental effect across The Bahamas.

Our primary analysis of male coloration comprised an investigation into the shared and unique patterns of phenotypic divergence between fragmentation regimes across the three *Gambusia* species (Fig.1D). To accomplish this, we conducted a mixed-model nested mancova using five coloration measures as dependent variables: average dorsal-fin redness (hereafter *D*_RG_), number of dorsal-fin spots, number of caudal-fin spots, and number of lateral body spots (all counts square-root transformed). Independent variables included BL (log-transformed size covariate), fragmentation regime (testing for a shared effect of fragmentation), species (testing for differences among species), and the interaction between fragmentation and species (to test for species specific, unique, effects of fragmentation), while island nested within species and population served as random effects.

The model was designed to test whether divergent species-level evolutionary histories of each population affected their response to shared ecological changes precipitated by ecosystem fragmentation. ‘Island’ and ‘population’ were random effects in our model to account for variation among replicate populations and because we wished to treat islands as random replicates for each species in order to focus on fragmentation effects across the three species. Random effects were included in mancova using proc MIXED in SAS (SAS Institute, Cary, NC, USA). Details on this analytical method are found in Hassell et al. (2012) and Riesch et al. (2013). Significance tests for fragmentation, species, and their interaction were conducted with *F*-tests using restricted maximum likelihood and Kenward–Roger degrees of freedom adjustment (Kenward and Roger 1997). Remaining analyses were conducted in JMP (SAS) and Wilks’s partial *η*^2^ was used to estimate the relative importance of model terms (see Langerhans and DeWitt 2004). We tested for heterogeneity of slopes among species, and between fragmentation regimes, by including BL in interaction terms with fixed main effects (Fragmentation and Species) and their interaction (Fragmentation × Species). Due to nonsignificance, we omitted these from our final mancova analysis.

To interpret and visualize morphological divergence due to fragmentation, we (i) examined univariate patterns for each trait and (ii) derived canonical variates from independent variables in our mancova model. First, we conducted separate analyses of variation for each phenotypic trait employing a univariate analog of the shared and unique model structure used in our mancova analysis. Second, we examined patterns along canonical variates and inspected canonical loadings for each variate (pairwise correlations of traits and variates). These canonical loadings indicate the relative influence of each coloration variable on phenotypic divergence across factor levels (Fragmentation, Species, and Fragmentation × Species). Each canonical variate describes the multivariate linear combination of coloration variables that maximize differences between groups while minimizing differences within groups. To evaluate significant differences between fragmentation regimes within each species along the shared and unique phenotypic axes (canonical variates derived from ‘Fragmentation’ and ‘Fragmentation × Species’ terms in our mancova, respectively), we conducted planned contrasts separately for each axis, using population as the unit of replication. Finally, to identify proximate relationships between environmental drivers and fragmentation-associated phenotypic divergence, we conducted a series of linear mixed models using population means of the shared and unique canonical variates derived from our mixed-model mancova as dependent variables and environmental variables as independent variables. We tested for main effects of environmental color (*E*_RG_ and *E*_GB_), turbidity, and piscivore density on major axes of phenotypic variation. We explored pairwise interactions between piscivore density and other fixed terms in the model to examine possible predator-dependent effects of the spectral environment. However, all interactions were nonsignificant and therefore excluded from the final model. Species, and Island nested within Species (random term), were included to control for historical effects across species and islands.

## Results

manova analysis of the effects of fragmentation on putative environmental drivers of phenotypic variation showed that tidal creek environments differed strongly according to fragmentation regime. We also detected environmental variation among the geographic ranges of each species (Table1), although the effects of fragmentation on environmental variables were highly consistent across the three species ranges as indicated by the nonsignificant interaction term (Table1). Wilks’s partial *η*^2^ indicated that the majority of environmental variation was attributable to fragmentation regime rather than geography (i.e., a shared effect of fragmentation on environmental attributes within each species range; Table1). Inspection of canonical variate loadings revealed that fragmentation was correlated with a reduction in piscivore density, a shift toward a more yellow/green spectral environment, and moderately increased turbidity (Table S3). Canonical loadings also suggest that the allopatric geographic ranges inhabited by each species differed in turbidity, environmental redness, and piscivore density. However, further analysis suggested that these differences were minor. Pairwise multivariate contrasts between species revealed that only the ranges of *Gambusia* sp. and *G. hubbsi* differed in measured environmental factors (Wilks’s = 0.59, *F*_4,38_ = 5.6, *P *=* *0.001) and follow-up univariate tests and pairwise comparisons (Tukey’s HSD) among species revealed that only turbidity, not environmental coloration or predator density, differed significantly among species ranges, with *G. hubbsi* inhabiting more turbid waters than *Gambusia* sp. (species means: *Gambusia* sp. = 0.48, *G. hubbsi =* 0.90, *G. manni* = 0.65).

**Table 1:** manova results for the effects of tidal creek fragmentation and geography on environmental conditions in 47 tidal creeks in The Bahamas.

Effect	*df*	*F*	*P*	*Wilks’s λ*	*Partial η* ^2^
Species range (SR)	8,76	2.7	0.013	0.61	0.22
Fragmentation (F)	4,38	3.5	0.015	0.37	0.63
F × SR	8,76	0.8	0.645	0.86	0.07

In our analysis of male *Gambusia* coloration, we found all factors in our mixed-model mancova exhibited highly significant effects (Table2). Coloration was strongly affected by body size. The shared ecological disturbance, fragmentation, was clearly the most important term in our model other than body size, explaining a large portion of the observed phenotypic variation as reflected by partial *η*^2^. Species, our historical variable, and the interaction between species and fragmentation, both explained less variance than the shared effect of fragmentation (Table2). All species showed similar trajectories of phenotypic divergence along the shared axis; however, differences between fragmented and unfragmented populations were not significant for all species (*Gambusia* sp., *t* = −1.5, *P* = 0.129; *G. hubbsi, t* = −0.5, *P* = 0.622; *G. manni*,*t* = −4.9, *P* < 0.001). Significant divergence along the first unique phenotypic axis also differed by species (*Gambusia*sp. *t* = 3.4, *P* = 0.001; *G. hubbsi*,*t* = 1.1, *P* = 0.279; *G. manni*,*t* = −6.7, *P* < 0.001). Finally, only one species differed along the second unique axis (*Gambusia*sp. *t* = −2.9, *P* = 0.005; *G. hubbsi*,*t* = 0.0, *P* = 0.997; *G. manni*,*t* = −1.8, *P* = 0.074).

**Table 2:** Mixed-model mancova results examining variation in coloration in male *Gambusia*collected from a series of fragmented and unfragmented tidal creeks in The Bahamas.

Effect	*df*	*F*	*P*	Wilks’s *λ*	Partial *η*^2^
Body length (BL)	3,457	23.9	<0.001	0.28	0.72
Species (S)	6,557	17.3	<0.001	0.47	0.31
Fragmentation (F)	3,457	4.7	0.003	0.13	0.87
F × S	6,557	5.1	<0.001	0.73	0.15

**Figure 3:**
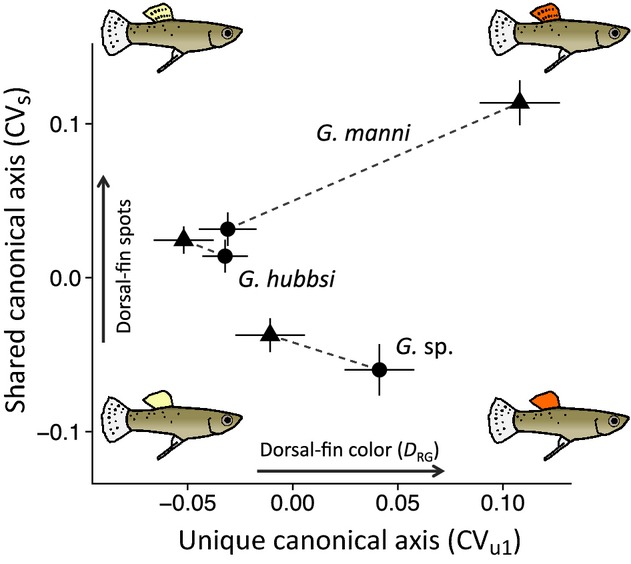
Shared and unique patterns of variation in male coloration among Bahamian mosquitofish species. Canonical axes, derived from our mancova model, show that all species tend to decrease in the number of dorsal-fin spots in fragmented sites (shared divergence), but exhibit species-specific shifts in fin coloration due to fragmentation (unique divergence). Points are species-specific canonical variate means ± standard errors; fragmented (•), unfragmented (▴).

Examination of canonical loadings revealed that all four traits (*D*_RG_, caudal-fin spots, dorsal-fin spots, and lateral spots) were strongly correlated with one or more canonical axes (Table S4). Loadings for body size (BL) were positive and large for both dorsal-fin spots and caudal-fin spots (loadings = 0.63 and 0.94, respectively), indicating an overall increase in these trait values with the size of fish. The first canonical variate derived from the species term, the historical variable, was strongly correlated with dorsal-fin spots (loading = −0.81) and lateral spotting (loading = 0.50), while the second canonical variate was strongly correlated with dorsal-fin coloration (loading = 0.91). These results indicated that species differences in male coloration can be roughly characterized as: *Gambusia*sp. tend to have reddish-orange dorsal fins (high *D*_RG_ values) with relatively few dorsal-fin spots, and high numbers of lateral spots; *G. hubbsi*have yellow-orange dorsal fins (low *D*_RG_ values) with moderate numbers of dorsal-fin, and lateral spots; *G. manni*also has reddish-orange dorsal fins (high *D*_RG_ values) with large numbers of dorsal-fin spots, and few lateral spots (Figure S3). Among shared and unique responses, fragmentation, the shared environmental factor, drove a strong shift in dorsal-fin spot number (loading = 0.79) reflecting a general pattern of reduced dorsal-fin spotting in fragmented populations, regardless of species identity (Fig.3, Table S4). Unique axes of phenotypic divergence were correlated with fin coloration and dorsal-fin spots. The first unique canonical axis explained variation in fin coloration (*D*_RG_ loading = 0.89). This reflected a pattern whereby species exhibited qualitatively different responses to fragmentation with respect to dorsal-fin color (Fig.3). The second unique axis explained variation in coloration (*D*_RG_ loading = −0.56) and dorsal-fin spotting (loading = 0.83); however, the amount of interaction variance explained by the second unique axis was small (<5%) and we do not consider it further.

Univariate analyses mirrored our mancova results; the effect of fragmentation on fin coloration depends on species identity while an overall effect of fragmentation on dorsal-fin spotting is evident although only nearly significant in the univariate analysis (*P* = 0.074; Tables S5 and S6). Overall, the three species exhibited the following phenotypic patterns across fragmentation regimes: *Gambusia*sp. exhibited little-to-no difference in dorsal-fin spotting and redder dorsal-fin coloration (high *D*_RG_ values) in fragmented sites; *G. hubbsi*were similar in dorsal-fin spotting and fin coloration among fragmented and unfragmented sites; *G. manni*exhibited fewer dorsal-fin spots and more yellow dorsal-fin coloration (low *D*_RG_ values) in fragmented sites.

Among environmental variables, turbidity and water coloration exhibited substantial evidence as drivers of phenotypic divergence (Table3). Fin coloration showed evidence of a negative effect for turbidity and a positive association with environmental redness (*E*_RG_). Turbidity was also negatively correlated with dorsal-fin spots although the relationship was merely suggestive (*P* = 0.097). Finally, environmental blueness (*E*_GB_) and piscivore density failed to show any effect on the traits examined (Table3).

**Table 3:** Summary of univariate mixed-models examining the associations between population-level environmental variation and phenotypic divergence axes from the mixed-model mancova (bold text emphasizing significant relationships).

Divergence axis	Source	Estimate	*df*	*F*	*P*
Shared CV_S_ (Dorsal-fin spots)	*E*_RG_	−0.25	1,40	1.8	0.187
	*E*_GB_	−0.06	1,40	0.6	0.435
	Predator	−0.00	1,14	0.0	0.844
	**Turbidity**	**−0.05**	**1,40**	**2.9**	**0.097**
	**Species**		**2,2**	**26.0**	**0.022**
Unique CV_U1_ (*D*_RG_)	***E***_**RG**_	**0.59**	**1,38**	**5.0**	**0.031**
	*E*_GB_	0.02	1,39	0.0	0.883
	Predator	−0.00	1,40	0.0	0.827
	**Turbidity**	**−0.13**	**1,38**	**8.1**	**0.007**
	Species		2,4	0.6	0.603

## Discussion

We document evidence for human-mediated phenotypic divergence among wild populations of Bahamian mosquitofish. This divergence exhibits evidence of both shared and unique responses among the three species. Below we discuss our evidence for parallel and nonparallel phenotypic divergence, putative mechanisms driving the observed divergence, and the implications of anthropogenically mediated change for the ecological and evolutionary futures of wild populations.

### Human-mediated divergence

Hydrologic fragmentation of coastal wetlands across the Bahama Archipelago has driven replicated patterns of morphological divergence among three, closely related, species. While restricted to dorsal-fin spots, this shared, divergent response is consistent with our initial expectation that the drastic environmental changes associated with tidal creek fragmentation affects coloration consistently, among, and within species. Proffering an adaptive scenario for the reduction of melanic spotting of dorsal fins in mosquitofish occupying fragmented habitats is difficult without a better understanding of their function (crypsis, signaling, both, or other). However, given the negative correlation between dorsal-fin spots and turbidity suggested by our univariate analysis, reduction in fin spots due to fragmentation may reflect a consequence of reduced effectiveness of visual signals in turbid environments. Melanic patterns have been hypothesized to increase the conspicuity of color signals by increasing contrast between monochromatic backgrounds and the focal signal feature (Hailman 1979). Therefore, if turbidity relaxes positive selection on contrast, we might expect this signal modifier to fade or become less elaborate over time. Indeed, such a response has been documented in other study systems, lending support to this explanation (Victoria cichlids: Seehausen et al. 1997; Maan et al. 2010; blue-fin killifish: Fuller 2002). Regardless of the mechanism, which requires future investigation, we uncovered strong evidence for a shared decrease in dorsal-fin spotting due to fragmentation in Bahamian mosquitofish (Fig.3, Table2).

Despite the similarity of environmental shifts induced by fragmentation and the recent evolutionary differentiation of these species (1–5 mya, Heinen-Kay et al. 2014), results also revealed an unexpectedly strong signature of species-contingent phenotypic divergence due to anthropogenic habitat alteration: Dorsal-fin coloration in *Gambusia*sp. and *G. manni*differed between fragmentation regimes (in opposite manners), while *G. hubbsi*exhibited no such differentiation (Fig.3). Below we discuss two questions that emerge from these results.

Why did fragmentation not influence dorsal-fin color in *G. hubbsi*? This finding was unexpected, especially in light of previous research from Andros Island blue holes showing that *G. hubbsi*fin coloration has adaptively diverged repeatedly between predation regimes (e.g., Langerhans et al. 2007; Langerhans 2010; Martin et al. 2014). One line of evidence points toward dorsal-fin color being a less important sexual signal within tidal creeks for *G. hubbsi*compared to the other two species. While the effect of fragmentation on tidal creek environments is highly consistent across the study area (Table1), habitats within the range of *G. hubbsi*are relatively turbid*,*regardless of fragmentation regime (Table S2). Perhaps signal transmission of orange dorsal fins is generally poor in these turbid tidal creeks, with fragmentation having an effectively small influence on this signal’s transmission environment. Future work is needed to test this hypothesis.

What generated dissimilar divergence trajectories in *G. manni*and *Gambusia*sp. when selective environments appeared similar? Explanations of divergent responses to similar selection regimes typically rely on cryptic variation in selection regimes (e.g., sensory drive and female preference), differences in genetic architecture, or the chance order of appearance of beneficial mutations in different lineages (Langerhans and Riesch 2013; Mendelson et al. 2014). If the fitness landscape is similarly rugged within *G. manni*and *Gambusia*sp, then the latter two mechanisms could explain divergence in the face of similar selection – because multiple adaptive solutions to environmental constraints on signal transmission exist. That is, one adaptive strategy to mitigate signal disruption is to increase signal investment (increased red color: *D*_RG_), presumably increasing transmission distance and the probability of accurate reception (e.g., guppies: Endler and Houde 1995; perch: Kekäläinen et al. 2010; pygmy perch: Morrongiello et al. 2010; rainbowfish: Kelley et al. 2012; shiners: Dugas and Franssen 2011; stickleback: Reimchen 1989). Such a compensatory response might resemble the increased fin redness of *Gambusia*sp. populations inhabiting fragmented, turbid habitats (Fig.3). Another strategy of potentially equal adaptive value might be to reduce signaling investment in antagonistic environments to minimize costs of ineffective signals (Järvenpää and Lindström 2004; Heuschele et al. 2009). This would be expected if fitness benefits accrued through restraining signal investment are similar to or greater than that accrued through increasing signal effort. Redirecting energy or pigments to more effective signals or viability could adaptively recover negative fitness consequences of reduced signaling effectiveness. Such a strategy might explain the general pattern of reduced dorsal-fin spotting in fragmented populations, the overall weakly colored, yellow dorsal fins in the turbid tidal creeks inhabited by *G. hubbsi*, and the reduced dorsal-fin redness within fragmented tidal creeks in *G. manni*(Fig.3). Evidence for such a strategy in the literature is not as strong; however, a few examples do illustrate a negative relationship between signal intensity and turbidity (Victoria cichlids: Seehausen et al. 1997; Maan et al. 2010; blue-fin killifish: Fuller 2002).

Overall, both shared and unique patterns of divergent phenotypic responses among *Gambusia*species to fragmentation suggest that sexual signals are shifting due to changes to the spectral environment rather than predation risk. While compelling, these findings leave many questions unanswered. For example, what aspects of genome architecture differ among our study species? What are the contributions of genetic and environmental sources to phenotypic responses? And are the environmental shifts experienced by each species similar enough to be considered ‘shared’? We have simplified our analysis of environmental drivers by limiting the set of environmental factors to predation and spectral environment, yet fragmentation precipitates a range of ecological changes. One potentially important variable we do not address here is geographic variation in the effect of fragmentation on salinity. The Bahama Archipelago shows a strong north–south precipitation gradient (Figure S4) causing hydrologically isolated ecosystems, such as fragmented tidal creeks, to become hyposaline in the north and hypersaline in the south (Figure S5). We suspect this apparent interaction between geography and fragmentation might indirectly affect other factors important for signal production such as the types and quantity of carotenoids in mosquitofish prey (Cifuentes et al. 2001; Oren 2005). Such variation in carotenoid availability could underlie the unique fin coloration responses to fragmentation we observe in our dataset (Grether et al. 1999; Craig and Foote 2001; Grether 2005) and deserves further study.

### Conservation implications

At least half of the world’s major river drainages are dammed (Nilsson et al. 2005), and a large number of smaller drainages are heavily affected by hydrological fragmentation. For example, in the United States alone, 2.6 million impoundments account for an estimated 20% of existing inland standing waters by area (Smith et al. 2002). Fragmentation impacts to coastal habitats are also frequent and heavy: even in the extensive marshlands of the southeastern United States, fragmentation directly affects approximately 84 000 ha (20%) of coastal wetlands (Montague et al. 1987). Damming is frequently cited as one of the most detrimental anthropogenic threats to freshwater faunal diversity, having extremely deleterious effects on endemic and anadromous species, especially fishes and invertebrates (March et al. 2003; Poff et al. 2007; Liermann et al. 2012; Cooney and Kwak 2013). Despite the global extent and huge ecological impacts of hydrologic disruption on biodiversity the number of studies investigating its impacts from an evolutionary perspective are remarkably few (e.g., Franssen 2011; Aguirre et al. 2013; Franssen et al. 2013a,b; Cureton and Broughton 2014; Heinen-Kay et al. 2014; Santos and Araújo 2015). In each of these studies, species persisting in altered habitats show significant phenotypic change. Typically, trait shifts are related to viability (e.g., locomotion and foraging), but changes in sexual traits are also apparent (Heinen-Kay et al. 2014). In concert, these findings inform a general argument that hydrologic fragmentation and the novel ecosystems generated as a consequence drives widespread evolutionary divergence in a variety of wild fish populations.

Predicting the fate of wild populations subjected to such a pervasive ecological disturbance is perhaps the most pressing conservation challenge (Rice and Emery 2003; Stockwell et al. 2003; Carroll and Fox 2008; Hendry et al. 2011; Carroll et al. 2014; Smith et al. 2014). Yet, as our results and others show (e.g., Franssen et al. 2013a), the direction and magnitude of phenotypic divergence may be difficult to predict, even when the focal species are closely related, ecologically similar, and subjected to seemingly similar selection regimes. These general conclusions, formed from this and other research exploring diverse responses to selection regimes (e.g., Kaeuffer et al. 2012) suggest a strong tendency for populations to diverge in parallel and nonparallel ways due to historically contingent factors. This fact complicates actions directed toward understanding and managing human-mediated evolutionary change in the wild (Carroll et al. 2014). Adequately capturing the predictably of phenotypic change required for evolutionarily informed conservation actions will require well-replicated studies of broad taxonomic, ecological, and geographic scope to uncover historically contingent influences on evolutionary trajectories.

## Conclusion

Sexual signals play an important role in a diversity of biological functions and evolutionary processes (Smith and Grether 2008). Habitat alteration poses a significant threat to organisms because it can disrupt signal transmission during mate evaluation (e.g., Järvenpää and Lindström 2004; Engström-Öst and Candolin 2006; Candolin et al. 2007) and species recognition (e.g., Seehausen et al. 1997; Fisher et al. 2006; Walters et al. 2008). Consequently, habitat alteration represents an important threat to a diversity of species that implement chemical, auditory, electrical, and visual signals in their communication systems (van der Sluijs et al. 2011; Rosenthal and Stuart-Fox 2012). Yet, the rapidity with which sexually selected traits can evolve (Endler 1986; Hoekstra et al. 2001; Kingsolver et al. 2001; Svensson and Gosden 2007), and the pervasiveness of anthropogenic environmental alteration, suggests that human-mediated divergence of sexual signaling systems is common among species that persist in altered ecosystems. Consequently, the future of wild populations may not depend on whether phenotypic change will occur. Rather, their fates will rely on whether the trajectory and magnitude of change is sufficient to ameliorate phenotype–environment mismatches and contribute to population persistence (Bell and Collins 2008; Barrett and Hendry 2012; Carroll et al. 2014; Zimova et al. 2014).

## Data archiving statement

Data for this study are available from the Dryad Digital Repository: http://dx.doi.org/10.5061/dryad.10b95.

## Supporting Information

Figure S1. Male Bahamian mosquitofish (*Gambusia*spp.) show marked interpopulation variation in dorsal fin coloration, as well as spotting on fins and flanks. **Figure S2.** Map of the upper Bahama Archipelago (inset) and its position in relation to the US and Greater Antilles. **Figure S3.** Differences in male coloration among Bahamian mosquitofish species. **Figure S4.** Latitudinal precipitation gradient in the Bahama Archipelago. **Figure S5.** The relationships between latitude and salinity in fragmented and unfragmented tidal creeks. **Table S1.** Repeatability analysis of environmental measures for a subset of fragmented and unfragmented tidal creeks on Abaco Island measured between 2009 and 2012. **Table S2.** Untransformed means for *Gambusia*morphology and environmental variables among fragmented and unfragmented habitats. **Table S3.** Loadings of environmental variables on canonical axes derived from MANOVA of effect of fragmentation and geography. **Table S4.** Loadings of canonical variates derived from the Allometric (SL), Species (Historical), Fragmentation Regime (Shared), and Species × Fragmentation Regime (Unique) terms of the mixed-model MANCOVA examining variation in male Bahamian *Gambusia*coloration (important loadings bolded for emphasis). **Table S5.** Summary results of shared and unique effects of fragmentation on phenotypic traits in Bahamian mosquitofish (*Gambusia*spp.). **Table S6.** Least-squares means and standard errors for each significant term in our univariate analysis of shared and unique effects of fragmentation.

## References

[b1] Aguirre WE, Shervette VR, Navarrete R, Calle P, Agorastos S (2013). Morphological and genetic divergence of *Hoplias microlepis*(Characiformes, Erythrinidae) in western Ecuador. Copeia.

[b2] Allgeier JA, Rosemond AD, Mehring AS, Layman CA (2010). Synergistic nutrient co-limitation across a gradient of ecosystem fragmentation in subtropical mangrove-dominated wetlands. Limnology and Oceanography.

[b3] Andersson M (1994). Sexual Selection.

[b4] Araújo MS, Langerhans RB, Giery ST, Layman CA (2014). Ecosystem fragmentation drives increased diet variation in an endemic livebearing fish of The Bahamas. Ecology and Evolution.

[b5] Archer SN, Endler JA, Lythgoe JN, Partridge JC (1987). Visual pigment polymorphism in the guppy *Poecilia reticulata*. Vision Research.

[b6] Barnosky AD, Matzke N, Tomiya S, Wogan GOU, Swartz B, Quental TB, Marshall C (2011). Has the Earth’s sixth mass extinction already arrived?. Nature.

[b7] Barrett RDH, Wong BBM, Hendry AP, Candolin U (2012). Evolutionary rescue. Behavioral Responses to a Changing World.

[b8] Bell G, Collins S (2008). Adaptation, extinction and global change. Evolutionary Applications.

[b9] Blount ZD, Borland CZ, Lenski RE (2008). Historical contingency and the evolution of a key innovation in an experimental population of *Escherichia coli*. Proceedings of the National Academy of Sciences of the United States of America.

[b10] Brooks R, Caithness N (1995). Female choice in a feral guppy population: are there multiple cues?. Animal Behaviour.

[b11] Candolin U, Salesto T, Evers M (2007). Changed environmental conditions weaken sexual selection in Sticklebacks. Journal of Evolutionary Biology.

[b12] Carroll SP, Fox CW (2008). Conservation Biology: Evolution in Action.

[b13] Carroll SP, Jørgensen PS, Kinnison MT, Bergstrom CT, Denison RF, Gluckman P, Smith TB (2014). Applying evolutionary biology to address global challenges. Science.

[b14] Chacin DH, Giery ST, Yeager L, Layman CA, Langerhans RB (2015). Does hydrological fragmentation affect coastal bird communities? A study from Abaco Island, The Bahamas. Wetlands Ecology and Management.

[b15] Cifuentes AS, González MA, Inostroza I, Aguilera A (2001). Reappraisal of physiological attributes of nine strains of *Dunaliella*(Chlorophyceae): growth and pigment content across a salinity gradient. Journal of Phycology.

[b16] Cooney PB, Kwak TJ (2013). Spatial extent and dynamics of dam impacts on tropical island freshwater fish assemblages. BioScience.

[b17] Craig JK, Foote CJ (2001). Countergradient variation and secondary sexual color: phenotypic convergence promotes genetic divergence in carotenoid use between sympatric anadromous and nonanadromous morphs of Sockeye Salmon. Evolution.

[b18] Cureton JC, Broughton RE (2014). Rapid morphological divergence of a stream fish in response to changes in water flow. Biology Letters.

[b19] Dawson TP, Jackson ST, House JI, Prentice IC, Mace GM (2011). Beyond predictions: biodiversity conservation in a changing climate. Science.

[b20] Dirzo R, Raven PH (2003). Global state of biodiversity and loss. Annual Review of Environment and Resources.

[b21] Downhower JF, Brown LP, Matsui ML (2000). Life history variation in female *Gambusia hubbsi*. Environmental Biology of Fishes.

[b22] Dugas MB, Franssen NR (2011). Nuptial coloration of red shiners (*Cyprinella lutrensis*) is more intense in turbid habitats. Naturwissenshaften.

[b23] Endler JA (1980). Natural selection on color patterns in *Poecilia reticulata*. Evolution.

[b24] Endler JA (1983). Natural and sexual selection on color patterns in Poeciliid fishes. Environmental Biology of Fishes.

[b25] Endler JA (1986). Natural Selection in the Wild.

[b26] Endler JA (1990). On the measurement and classification of colour in studies of animal colour patterns. Biological Journal of the Linnean Society.

[b27] Endler JA (1991). Variation in the appearance of guppy color patterns to guppies and their predators under different visual conditions. Vision Research.

[b28] Endler JA (2012). A framework for analyzing colour pattern geometry: adjacent colours. Biological Journal of the Linnean Society.

[b29] Endler JA, Houde AE (1995). Geographic variation in female preferences for male traits in *Poecilia reticulata*. Evolution.

[b30] Engström-Öst J, Candolin U (2006). Human-induced water turbidity alters selection on sexual displays in sticklebacks. Behavioral Ecology.

[b31] Faunce CH, Serafy JE (2007). Nearshore habitat use by gray snapper (*Lutjanus griseus*) and bluestriped grunt (*Haemulon sciurus*): environmental gradients and ontogenetic shifts. Bulletin of Marine Science.

[b32] Fisher RA (1930). The Genetical Theory of Natural Selection.

[b33] Fisher HS, Wong BBM, Rosenthal GG (2006). Alteration of the chemical environment disrupts communication in a freshwater fish. Proceedings of the Royal Society B – Biological Sciences.

[b34] Franssen NR (2011). Anthropogenic habitat alteration induced rapid morphological divergence in a native stream fish. Evolutionary Applications.

[b35] Franssen NR, Harris J, Clark SR, Schaefer JF, Stewart LK (2013a). Shared and unique morphological responses of stream fishes to anthropogenic habitat alteration. Proceedings of the Royal Society B-Biological Sciences.

[b36] Franssen NR, Stewart LK, Schaefer JF (2013b). Morphological divergence and flow-induced phenotypic plasticity in a native fish from anthropogenically altered stream habitats. Ecology and Evolution.

[b37] Fuller RC (2002). Lighting environment predicts the relative abundance of male colour morphs in bluefin killifish (*Lucania goodei*) populations. Proceedings of the Royal Society B-Biological Sciences.

[b38] Giery ST, Layman CA (2015). Interpopulation variation in a condition-dependent signal: predation regime affects signal intensity and reliability. American Naturalist.

[b39] Grether GF (2005). Environmental change, phenotypic plasticity and genetic compensation. American Naturalist.

[b40] Grether GF, Hudon J, Millie DF (1999). Carotenoid limitation of sexual coloration along an environmental gradient in guppies. Proceedings of the Royal Society B-Biological Sciences.

[b41] Gould SJ (1989). Wonderful Life: The Burgess Shale and the Nature of History.

[b42] Gould SJ, Lewontin R (1979). The spandrels of San Marco and the Panglossian paradigm: a critique of the adaptationist programme. Proceedings of the Royal Society B-Biological Sciences.

[b43] Hailman JP (1979). Optical Signals: Animal Communication and Light.

[b44] Hassell EMA, Meyers PJ, Billman EJ, Rasmussen JE, Belk MC (2012). Ontogeny and sex alter the effect of predation on body shape in a livebearing fish: sexual dimorphism, parallelism, and costs of reproduction. Ecology and Evolution.

[b45] Heinen-Kay JL, Noel HG, Layman CA, Langerhans RB (2014). Human-caused habitat fragmentation can drive rapid divergence of male genitalia. Evolutionary Applications.

[b46] Heinen-Kay JL, Morris KE, Ryan NA, Byerley SL, Venezia RE, Peterson MN, Langerhans RB (2015). A tradeoff between natural and sexual selection underlies diversification of a sexual signal. Behavioral Ecology.

[b47] Hendry AP, Farrugia TJ, Kinnison MT (2008). Human influences on rates of phenotypic change in wild animal populations. Molecular Ecology.

[b48] Hendry AP, Kinnison MT, Heino M, Day T, Smith TB, Fitt G, Bergstrom CT (2011). Evolutionary principles and their practical application. Evolutionary Applications.

[b49] Heuschele J, Mannerla M, Gienapp P, Candolin U (2009). Environment-dependent use of mate choice cues in sticklebacks. Behavioral Ecology.

[b50] Hoekstra HE, Hoekstra JM, Berrigan D, Vignieri SN, Hoang A, Hill CE, Beerli P (2001). Strength and tempo of directional selection in the wild. Proceedings of the National Academy of Sciences of the United States of America.

[b51] Horth L (2003). Melanic body colour and aggressive mating behaviour are correlated traits in male mosquitofish (*Gambusia holbrooki*. Proceedings of the Royal Society B-Biological Sciences.

[b52] Horth L (2004). Predation and the persistence of melanic male mosquitofish (*Gambusia holbrooki*. Journal of Evolutionary Biology.

[b53] Houde AE (1997). Sex, Color, and Mate Choice in Guppies.

[b54] Järvenpää M, Lindström K (2004). Water turbidity by algal blooms causes mating system breakdown in a shallow-water fish, the sand goby *Pomatoschistus minutus*. Proceedings of the Royal Society B-Biological Sciences.

[b55] Kaeuffer R, Peichel CL, Bolnick DI, Hendry AP (2012). Parallel and nonparallel aspects of ecological, phenotypic, and genetic divergence across replicate population pairs of lake and stream stickleback. Evolution.

[b56] Kekäläinen J, Huuskonen H, Kiviniemi V, Taskinen J (2010). Visual conditions and habitat shape the coloration of the Eurasian perch (*Perca fluviatilis*L.): a trade-off between camouflage and communication?. Biological Journal of the Linnaean Society.

[b57] Kelley JL, Phillips B, Cummins GH, Shand J (2012). Changes in the visual environment affect colour signal brightness and shoaling behaviour in a freshwater fish. Animal Behaviour.

[b58] Kenward MG, Roger JH (1997). Small sample inference for fixed effects from restricted maximum likelihood. Biometrics.

[b59] Kingsolver JG, Hoekstra HE, Hoekstra JM, Berrigan D, Vignieri SN, Hill CE, Hoang A (2001). The strength of phenotypic selection in natural populations. American Naturalist.

[b60] Kocher TD, Conroy JA, McKaye KR, Stauffer JR (1993). Similar morphologies of cichlid fish in Lakes Tanganyika and Malawi are due to convergence. Molecular Phylogenetics and Evolution.

[b61] Körner KE, Schlupp I, Plath M, Loew ER (2006). Spectral sensitivity of mollies: 830 comparing surface- and cave-dwelling Atlantic mollies, *Poecilia mexicana*. Journal of Fish Biology.

[b62] Lande R (1981). Models of speciation by sexual selection on polygenic traits. Proceedings of the National Academy of Sciences of the United States of America.

[b63] Langerhans RB, Elewa AMT (2006). Evolutionary consequences of predation: avoidance, escape, reproduction, and diversification. Predation in Organisms: A Distinct Phenomenon.

[b64] Langerhans RB (2010). Predicting evolution with generalized models of divergent selection: a case study with poeciliid fish. Integrative and Comparative Biology.

[b65] Langerhans RB, DeWitt TJ (2004). Shared and unique features of evolutionary diversification. American Naturalist.

[b66] Langerhans RB, Riesch R (2013). Speciation by selection: a framework for understanding ecology’s role in speciation. Current Zoology.

[b67] Langerhans RB, Layman CA, DeWitt TJ (2005). Male genital size reflects a tradeoff between attracting mates and avoiding predators in two live-bearing fish species. Proceedings of the National Academy of Sciences of the United States of America.

[b68] Langerhans RB, Gifford ME, Joseph EO (2007). Ecological speciation in *Gambusia*fishes. Evolution.

[b69] Layman CA, Silliman BR (2002). Preliminary survey and diet analysis of juvenile fishes of an estuarine creek on Andros Island, Bahamas. Bulletin of Marine Science.

[b70] Layman CA, Arrington DA, Langerhans RB, Silliman BR (2004). Degree of fragmentation affects assemblage structure in Andros Island (Bahamas) estuaries. Caribbean Journal of Science.

[b71] Layman CA, Quattrochi JP, Peyer CM, Allgeier JE (2007). Niche width collapse in a resilient top predator following ecosystem fragmentation. Ecology Letters.

[b72] Lessells CM, Boag PT (1987). Unrepeatable repeatabilities - A common mistake. Auk.

[b73] Levine JS, MacNichol EF (1979). Visual pigments in teleost fish: effects of habitat, microhabitat and behaviour on visual system evolution. Sensory Processes.

[b74] Liermann CR, Nilsson C, Robertson J, Ng RY (2012). Implications of dam obstruction for global freshwater fish diversity. BioScience.

[b75] Loew ER, Lythgoe JN (1978). The ecology of cone pigments in teleost fishes. Vision Research.

[b76] Losos JB (2010). Adaptive radiation, ecological opportunity, and evolutionary determinism. American Naturalist.

[b77] Losos JB, Jackman TR, Larson A, de Queiroz K, Rodriguez-Schettino L (1998). Contingency and determinism in replicated adaptive radiations of island lizards. Science.

[b78] Lythgoe JN, Muntz WRA, Partridge JC, Shand J, Williams DMcB (1994). The ecology of the visual pigments of snappers (Lutjanidae) on the Great Barrier Reef. Journal of Comparative Physiology A, Sensory, Neural, and Behavioral Physiology.

[b79] Maan ME, Seehausen O, Van Alphen JJM (2010). Female mating preferences and male coloration covary with water transparency in a Lake Victoria cichlid fish. Proceedings of the Royal Society B-Biological Sciences.

[b80] Mahler DL, Ingram T, Revell LJ, Losos JB (2013). Exceptional convergence on the macroevolutionary landscape in island lizard radiations. Science.

[b81] Mani GS, Clarke BC (1990). Mutational order: a major stochastic process in evolution. Proceedings of the Royal Society B-Biological Sciences.

[b82] March JG, Benstead JP, Pringle CM, Scatena FN (2003). Damming tropical island streams: problems, solutions, and alternatives. BioScience.

[b83] Martin RA, Riesch R, Heinen-Kay JL, Langerhans RB (2014). Evolution of male coloration during a post-Pleistocene radiation of Bahamas mosquitofish (*Gambusia hubbsi*. Evolution.

[b84] Mayr E (1942). Systematics and the Origin of Species, from the Viewpoint of a Zoologist.

[b85] McKay BD (2013). The use of digital photography in systematics. Biological Journal of the Linnean Society.

[b86] Mendelson TC, Martin MD, Flaxman SM (2014). Mutation-order divergence by sexual selection: diversification of sexual signals in similar environments as a first step in speciation. Ecology Letters.

[b87] Montague CL, Alexander VZ, Percival HF (1987). Ecological effects of coastal marsh impoundments: a review. Environmental Management.

[b88] Morrongiello JR, Bond NR, Crook DA, Wong BBM (2010). Nuptial coloration varies with ambient light environment in a freshwater fish. Journal of Evolutionary Biology.

[b89] Nei M, Maruyama T, Chakraborty R (1975). The bottleneck effect and genetic variability of populations. Evolution.

[b90] Nilsson C, Reidy A, Dybesius M, Revenga C (2005). Fragmentation and flow regulation of the world’s large river systems. Science.

[b91] Oren A (2005). A hundred years of *Dunaliella*research: 1905–2005. Saline Systems.

[b92] Palkovacs EP, Kinnison MT, Correa C, Dalton CM, Hendry AP (2012). Fates beyond traits: ecological consequences of human-induced trait change. Evolutionary Applications.

[b93] Palumbi SR (2001). Humans as the world′s greatest evolutionary force. Science.

[b94] Poff NL, Olden JD, Merritt DM, Pepin DM (2007). Homogenization of regional river dynamics by dams and global biodiversity implications. Proceedings of the National Academy of Sciences of the United States of America.

[b95] Pringle CM (2001). Hydrological connectivity and the management of biological reserves: a global perspective. Ecology.

[b96] Reimchen TE (1989). Loss of nuptial color in threespine sticklebacks (*Gasterosteus aculeatus*. Evolution.

[b97] Reznick DN, Endler JA (1982). The impact of predation on life history evolution in Trinidadian guppies (*Poecilia reticulata*. Evolution.

[b98] Rice KJ, Emery NC (2003). Managing microevolution: restoration in the face of global change. Frontiers in Ecology and the Environment.

[b99] Riesch R, Martin RA, Langerhans RB (2013). Predation’s role in life-history evolution of a livebearing fish and a test of the Trexler-DeAngelis model of maternal provisioning. American Naturalist.

[b100] Rosenblum EB (2006). Convergent evolution and divergent selection: lizards at the White Sands ecotone. American Naturalist.

[b101] Rosenblum EB, Harmon LJ (2011). “Same same but different”: replicated ecological speciation at white sands. Evolution.

[b102] Rosenthal GG, Wong BBM, Stuart-Fox D, Candolin U (2012). Environmental disturbance and animal communication. Behavioral Responses to a Changing World.

[b103] Rundell RJ, Price TD (2009). Adaptive radiation, nonadaptive radiation, ecological speciation and nonecological speciation. Trends in Ecology and Evolution.

[b104] Santos ABI, Araújo FG (2015). Evidence of morphological differences between *Astyanax bimaculatus*(Actinopterygii: Characidae) from reaches above and below dams on a tropical river. Environmental Biology of Fishes.

[b105] Schluter D (1996). Adaptive radiation along genetic lines of least resistance. Evolution.

[b106] Schluter D (2009). Evidence for ecological speciation and its alternative. Science.

[b107] Schluter D, McPhail JD (1992). Ecological character displacement and speciation in sticklebacks. American Naturalist.

[b108] Schluter D, McPhail JD (1993). Character displacement and replicate adaptive radiation. Trends in Ecology and Evolution.

[b109] Schwartz AK, Hendry AP (2010). Testing the influence of local forest canopy clearing on phenotypic variation in Trinidadian guppies. Functional Ecology.

[b110] Seehausen O, Van Alphen JJM, Witte F (1997). Cichlid fish diversity threatened by eutrophication that curbs sexual selection. Science.

[b111] Sih A, Ferrari MCO, Harris DJ (2011). Evolution and behavioral responses to human induced rapid environmental change. Evolutionary Applications.

[b112] Smith SV, Renwick WH, Bartley JD, Buddemeir RD (2002). Distribution and significance of small, artificial water bodies across the United States landscape. Science of the Total Environment.

[b113] Smith TB, Fox CW, Grether GF, Carroll SP (2008). The importance of conserving evolutionary processes. Conservation Biology: Evolution in Action.

[b114] Smith TB, Kinnison MT, Strauss SY, Fuller TL, Carroll SP (2014). Prescriptive evolution to conserve and manage biodiversity. Annual Review of Ecology, Evolution, and Systematics.

[b115] Stevens M, Parraga CA, Cuthill IC, Partridge JC, Troscianko TS (2007). Using digital photography to study animal coloration. Biological Journal of the Linnaean Society.

[b116] Stockwell CA, Hendry AP, Kinnison MT (2003). Contemporary evolution meets conservation biology. Trends in Ecology and Evolution.

[b117] Svensson EI, Gosden TP (2007). Contemporary evolution of secondary sexual traits in the wild. Functional Ecology.

[b118] Taylor EB, McPhail JD (2000). Historical contingency and determinism interact to prime speciation in sticklebacks. Proceedings of the Royal Society B-Biological Sciences.

[b119] Travisano M, Mongold JA, Bennett AF, Lenski RE (1995). Experimental tests of the roles of adaptation, chance, and history in evolution. Science.

[b120] Valentine-Rose L, Cherry JA, Culp JJ, Perez KE, Pollock JB, Arrington DA, Layman CA (2007). Floral and faunal differences between fragmented and unfragmented Bahamian tidal creeks. Wetlands.

[b121] van der Sluijs I, Gray SM, Amorim MCP, Barber I, Candolin U, Hendry AP, Krahe R (2011). Communication in troubled waters response of fish communication systems to changing environments. Evolutionary Ecology.

[b122] Vitousek PM, Mooney H, Lubchenco J, Melillo JM (1997). Human domination of Earth′s ecosystems. Science.

[b123] Walters DM, Blum MJ, Rashleigh B, Freeman BJ, Porter BA, Burkhead NM (2008). Red shiner invasion and hybridization with blacktail shiner in the upper Coosa River, USA. Biological Invasions.

[b124] Watson CT, Lubieniecki KP, Loew E, Davidson WS, Breden F (2010). Genomic organization of duplicated short wave-sensitive and long wave-sensitive opsin genes in the green swordtail, *Xiphophorus helleri*. BMC Evolutionary Biology.

[b125] Zimova M, Mills LS, Lukacs PM, Mitchell MS (2014). Snowshoe hares display limited phenotypic plasticity to mismatch in seasonal camouflage. Proceedings of the Royal Society B-Biological Sciences.

