# Supplementary material for: Anthropogenic ecosystem fragmentation drives shared and unique patterns of sexual signal divergence among three species of Bahamian mosquitofish
Source: Evol Appl. 2015 Jul 16;8(7):679–91. doi: 10.1111/eva.12275 (PMC4516420; doi:10.1111/eva.12275)
Supplement: Figure S1. — Male Bahamian mosquitofish (Gambusiaspp.) show marked interpopulation variation in dorsal fin coloration, as well as spotting on fins and flanks. Figure S2. Map of the upper Bahama Archipelago (inset) and its position in relation to the US and Greater Antilles. Figure S3. Differences in male coloration among Bahamian mosquitofish species. Figure S4. Latitudinal precipitation gradient in the Bahama Archipelago. Figure S5. The relationships between latitude and salinity in fragmented and unfragmented tidal creeks. Table S1. Repeatability analysis of environmental measures for a subset of fragmented and unfragmented tidal creeks on Abaco Island measured between 2009 and 2012. Table S2. Untransformed means for Gambusiamorphology and environmental variables among fragmented and unfragmented habitats. Table S3. Loadings of environmental variables on canonical axes derived from MANOVA of effect of fragmentation and geography. Table S4. Loadings of canonical variates derived from the Allometric (SL), Species (Historical), Fragmentation Regime (Shared), and Species × Fragmentation Regime (Unique) terms of the mixed-model MANCOVA examining variation in male Bahamian Gambusiacoloration (important loadings bolded for emphasis). Table S5. Summary results of shared and unique effects of fragmentation on phenotypic traits in Bahamian mosquitofish (Gambusiaspp.). Table S6. Least-squares means and standard errors for each significant term in our univariate analysis of shared and unique effects of fragmentation. [file eva0008-0679-sd1.docx]

**SUPPLEMENTAL INFORMATION**

Table S1. Repeatability analysis of environmental measures for a subset of fragmented and unfragmented tidal creeks on Abaco Island measured in 2009, 2011, and 2012. The strength of the correlation between measures is indicated by the intraclass correlation coefficient, *r* (Lessells and Boag 1987).

| **Environmental Variable** | **Duration** | **Measures per Site** | **# Sites** | **Total Measures** | ***r*** | ***P*** |
| --- | --- | --- | --- | --- | --- | --- |
| *E*_RG_ | 3 yrs | 3 | 13 | 39 | 0.45 | 0.0010 |
| *E*_GB_ | 3 yrs | 3 | 13 | 39 | 0.58 | 0.0002 |
|  | | | | | | |

Table S2. Untransformed means for *Gambusia* morphology and environmental variables among fragmented (Frag) and unfragmented (Unfrag) habitats. Because of the positive linear relationship between body length and caudal-fin and dorsal-fin spots, we present size-corrected least-squares means for those variables.

|  |  | ***Gambusia sp.*** | | ***G. hubbsi*** | | ***G. manni*** | |
| --- | --- | --- | --- | --- | --- | --- | --- |
|  |  | **Frag** | **Unfrag** | **Frag** | **Unfrag** | **Frag** | **Unfrag** |
|  | n populations | 10 | 10 | 6 | 7 | 8 | 6 |
| Phenotypic Traits | Body Length | 2.4 | 2.3 | 2.3 | 2.3 | 2.5 | 2.4 |
|  | *D*_RG_ | 0.15 | 0.11 | 0.08 | 0.07 | 0.10 | 0.14 |
|  | Caudal-fin spots | 20.1 | 21.5 | 19.2 | 20.0 | 24.9 | 21.8 |
|  | Dorsal-fin spots | 9.2 | 10.6 | 12.1 | 12.2 | 12.6 | 15.0 |
|  | Lateral spots | 7.5 | 6.7 | 5.2 | 3.1 | 4.6 | 3.7 |
|  |  |  |  |  |  |  |  |
| Environmental Characters | *E*_RG_ | -0.01 | -0.05 | -0.13 | -2.70 | -0.01 | -0.03 |
|  | *E*_GB_ | 0.32 | 0.20 | 0.22 | 0.16 | 0.35 | 0.13 |
|  | Turbidity | 3.2 | 1.8 | 10.1 | 8.5 | 7.9 | 2.9 |
|  | Predator Abundance | 5.5 | 136.2 | 0.9 | 49.4 | 9.6 | 23.6 |

Table S3. Loadings of environmental variables on canonical axes derived from MANOVA of effect of fragmentation and geography. The axes represent the canonical variate derived from the Fragmentation term (CV_F_) and the two canonical variates derived from the Species Range term (CV_G#_; bolding added to emphasize important loadings).

|  | **Fragmentation** | **Species Range** | |
| --- | --- | --- | --- |
|  | **CV_F_** | **CV_G1_** | **CV_G2_** |
| *E*_RG_ | **-0.59** | 0.09 | **-0.54** |
| *E*_GB_ | **0.91** | -0.34 | -0.28 |
| Turbidity | **0.46** | **0.87** | 0.01 |
| Predator Abundance | **-0.53** | -0.27 | **0.88** |

Table S4. Loadings of canonical variates derived from the Allometric (BL), Species (Historical), Fragmentation Regime (Shared), and Species × Fragmentation Regime (Unique) terms of the mixed-model MANCOVA examining variation in male Bahamian *Gambusia* coloration (important loadings bolded for emphasis).

|  | **Allometry** | **Historical** | | **Shared** | **Unique** | |
| --- | --- | --- | --- | --- | --- | --- |
| **Morphology** | **CV_BL_** | **CV_H1_** | **CV_H2_** | **CV_S_** | **CV_U1_** | **CV_U2_** |
| *D*_RG_ | 0.19 | 0.48 | **0.91** | -0.05 | **0.89** | **-0.56** |
| Caudal-fin spots | **0.94** | -0.18 | 0.30 | 0.18 | -0.11 | 0.20 |
| Dorsal-fin spots | **0.63** | **-0.81** | 0.20 | **0.79** | 0.03 | **0.83** |
| Lateral spots | -0.09 | **0.50** | 0.02 | -0.48 | -0.08 | 0.18 |
| Eigenvalue | 0.28 | 0.67 | 0.26 | 0.13 | 0.35 | 0.02 |

Table S5. Summary results of shared and unique effects of fragmentation on phenotypic traits in Bahamian mosquitofish (*Gambusia* spp.). Linear mixed models in which Population, and Island nested within Species were random terms were employed in each model. Bold type highlights results of interest.

| **Morphological Variable** | **Source** | ***df*** | ***F*** | ***P*** |
| --- | --- | --- | --- | --- |
| *D*_RG_ | **Body Length (BL)** | **1,220** | **4.4** | **0.0380** |
|  | Species (S) | 2,3 | 1.9 | 0.2975 |
|  | Fragmentation (F) | 1,39 | 0.0 | 0.9495 |
|  | **F × S** | **2,39** | **3.7** | **0.0331** |
|  |  |  |  |  |
| Caudal-fin spots | **Body Length (BL)** | **1,207** | **70.1** | **<0.0001** |
|  | Species (S) | 2,.2 | 2.6 | 0.7623 |
|  | Fragmentation (F) | 1,40 | 0.0 | 0.8607 |
|  | F × S | 2,40 | 0.5 | 0.6228 |
|  |  |  |  |  |
| Dorsal-fin spots | **Body Length (BL)** | **1,207** | **17.0** | **<0.0001** |
|  | **Species (S)** | **2,3** | **6.5** | **0.0840** |
|  | **Fragmentation (F)** | **1,39** | **3.4** | **0.0741** |
|  | F × S | 2,39 | 0.8 | 0.4772 |
|  |  |  |  |  |
| Lateral spots | Body Length (BL) | 1,202 | 0.0 | 0.9239 |
|  | Species (S) | 2.4 | 2.8 | 0.5687 |
|  | Fragmentation (F) | 1,38 | 1.0 | 0.3190 |
|  | F × S | 2,38 | 0.2 | 0.8216 |

Table S6. Least-squares means and standard errors for each significant term in our univariate analysis of shared and unique effects of fragmentation.

|  |  | **Fragmented** | | **Unfragmented** | |
| --- | --- | --- | --- | --- | --- |
| **Species** | **Trait** | **LMS** | **SE** | **LMS** | **SE** |
| *G.* sp. | *D*_RG_ | 0.14 | 0.02 | 0.11 | 0.02 |
| *G. hubbsi* | *D*_RG_ | 0.09 | 0.02 | 0.07 | 0.02 |
| *G. manni* | *D*_RG_ | 0.10 | 0.02 | 0.14 | 0.02 |
| All | Dorsal-fin spots | 3.35 | 0.08 | 3.54 | 0.08 |


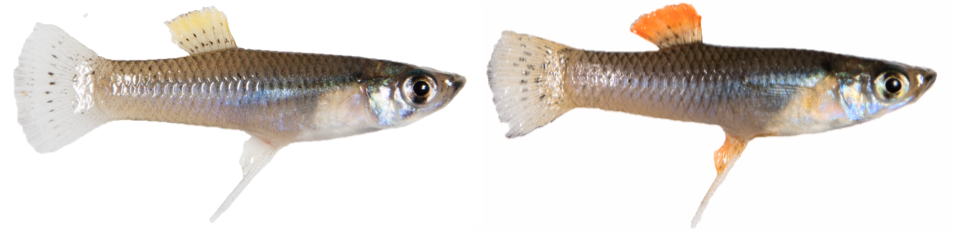


Figure S1. Male Bahamian mosquitofish (*Gambusia* spp.) show marked interpopulation variation in dorsal fin coloration, as well as spotting on fins and flanks. The individuals above are of an unnamed species from Abaco Island (*Gambusia* sp.). Their divergent phenotypes typify the effect of fragmentation on that species. On the left is an individual from an unfragmented site and on the right is an individual from a fragmented site showing the reddish-orange dorsal fins (high *D*_RG_) typical of individuals from fragmented sites in this species.


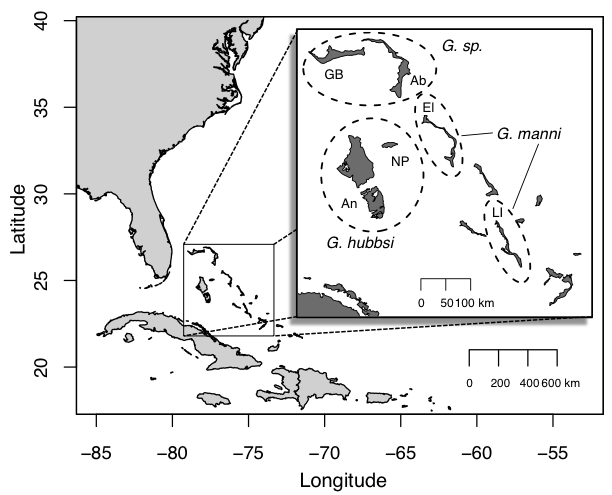


Figure S2. Map of the upper Bahama Archipelago (inset) and its position in relation to the US and Greater Antilles. The inset map shows the islands on which the three *Gambusia* species sampled for this study are located. Island names are: Grand Bahama (GB), Abaco (Ab), Eleuthera (El), Long Island (LI), New Providence (NP), Andros (An).


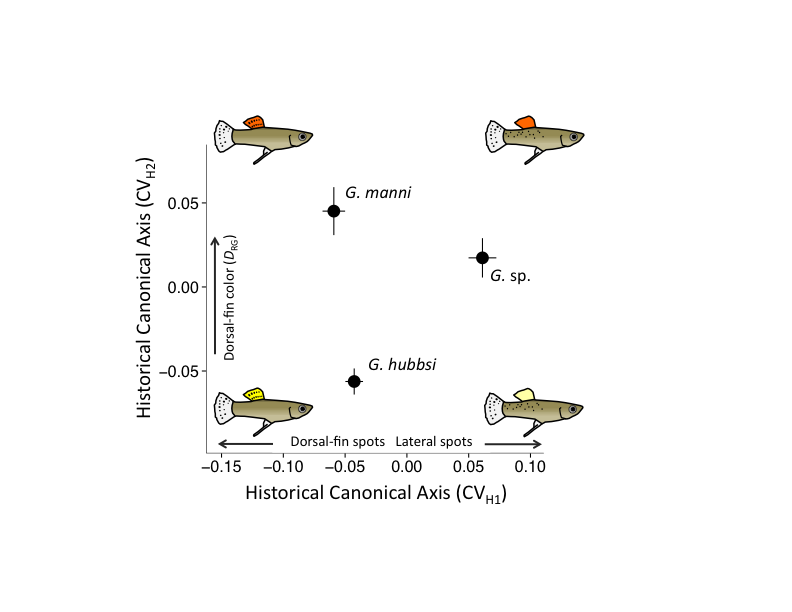


Figure S3. Differences in male coloration among Bahamian mosquitofish species. Canonical axes, derived from the species term in our MANCOVA model, show that species differ along two phenotypic axes: dorsal fin and lateral spotting, and the color of the dorsal fin. Data points represent species-specific canonical variate means ± standard errors.


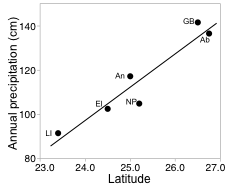


Figure S4. Latitudinal precipitation gradient in the Bahama Archipelago. Except New Providence, data are means of yearly totals between 1973 - 1990. NP data are annual means from 2000-2005. Data source: henge.bio.miami.edu/coastalecology/weather_and_climate_data.htm.


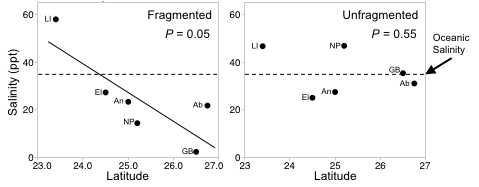


Figure S5. The relationships between latitude and salinity in fragmented and unfragmented tidal creeks. Average oceanic salinity (~35ppt) is displayed as dashed line. Latitude estimates were derived in Google Earth from approximate centers of islands in our analysis. Salinity measures were recorded during site visits in 2010.
